# Extended Subtotal Mandibulectomy for the Treatment of Oral Tumors Invading the Mandibular Canal in Dogs—A Novel Surgical Technique

**DOI:** 10.3389/fvets.2019.00339

**Published:** 2019-10-04

**Authors:** Nadine Fiani, Santiago Peralta

**Affiliations:** Department of Clinical Sciences, College of Veterinary Medicine, Cornell University, Ithaca, NY, United States

**Keywords:** mandibulectomy, extended subtotal mandibulectomy, mandibular canal, canine oral tumor, canine oral neoplasia

## Abstract

Mandibular tumors in the oral cavity of dogs can be locally aggressive and infiltrative, involving adjacent soft and hard tissues. Tumors that invade the mandibular canal are considered likely to extend rostrally and caudally within that structure due to minimal tissue resistance. When this occurs, a total mandibulectomy is thought to be the treatment of choice as it allows *en bloc* excision of the mandibular canal. This procedure is technically challenging and time consuming. In the present report we describe a novel technique, the extended subtotal mandibulectomy, as a possible alternative in cases of mandibular body tumors that have invaded the mandibular canal. This technique allows the complete excision of the mandibular canal whilst retaining the coronoid and condylar processes.

## Introduction

**Figure 1:**
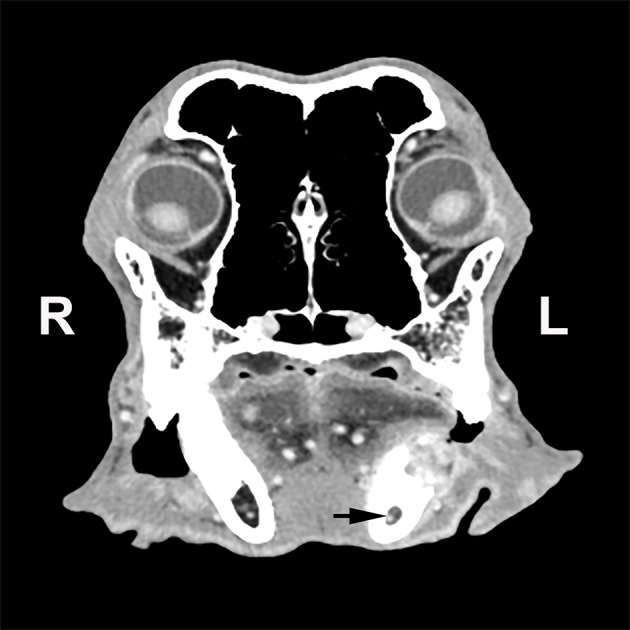
Transverse computed tomographic image of a dog with a left mandibular tumor at the level of the first molar tooth. The tumor has resulted in lysis of the dorsal aspect of the mandible and has extended into the mandibular canal (black arrow).

Oral neoplasms are common in dogs, representing ~6% of all tumors in this species (1, 2). The most common oral neoplasms in dogs include melanoma, squamous cell carcinoma, fibrosarcoma, canine acanthomatous ameloblastoma, and peripheral odontogenic fibroma (3, 4). Oral tumors can be locally aggressive and infiltrative involving adjacent soft and hard tissues (5). Depending on anatomical location, *en bloc* excision via maxillectomy or mandibulectomy is the treatment of choice for most oral tumors (2, 6–8). However, the precise surgical technique used and anatomical planes included are determined based on clinical, radiographic, and clinicopathological findings including type and size of the tumor (6, 9). Some of the mandibulectomy techniques and anatomical configurations described include unilateral rostral, bilateral rostral, segmental, marginal, caudal, and subtotal excisions (2, 6, 9). Total mandibulectomy is indicated when the tumor has invaded into the mandibular canal (6, 9) (Figure 1). The rationale is that the mandibular canal, which is located in the ventral third of the mandibular body and contains the inferior alveolar neurovascular bundle, offers minimal anatomical planes of resistance and tumors are likely to track along rostrally and caudally (9).

Due to the anatomical structures involved, a total mandibulectomy is the most invasive and technically demanding type of mandibulectomy. In contrast, subtotal mandibulectomy is a considerably less invasive procedure. However, the osteotomy is performed just caudal to the mandibular third molar tooth and thus does not allow excision of the mandibular canal in its entirety. Arguably, a surgical technique that would allow *en bloc* excision of the mandibular canal but would not require disarticulation of the temporomandibular joint and removal of the mandibular condylar and coronoid processes could be beneficial for the patient as it would minimize surgical morbidity and reduce anesthetic time. Here we describe a surgical technique that allows complete excision of the mandibular canal without requiring excision of coronoid and condylar processes, which we refer to as an *extended subtotal mandibulectomy*. Three clinical cases are reported to better illustrate its application followed by a brief discussion aimed at highlighting its merit.

## Surgical Technique

Following induction of general anesthesia, the oral cavity was irrigated with 0.12% chlorhexidine gluconate to minimize the bacterial burden. Supra- and subgingival ultrasonic scaling of all teeth was performed. The affected mandible was treated last to avoid dissemination of neoplastic cells to other areas of the mouth. An inferior alveolar regional nerve block was performed at the affected mandible. The ventral aspect of the mandible and the maxilla, from the level of the philtrum to the medial canthus of the eye, were surgically prepared for aseptic surgery.

Patients were placed in dorsal recumbency and surgically draped. A sterile surgical ruler and marker was used to measure and delineate the soft tissue margins intended for excision. An intraoral approach was used and soft tissue incisions were determined based on planned margins according to the type of tumor, diagnostic imaging, and oral examination findings (9). A number 15 scalpel blade was used to incise oral mucosal margins. A combination of sharp and blunt dissection of the soft tissues was performed until underlying mandibular bone was exposed. The (rostral, middle and caudal) mental foramina were identified, and the corresponding emerging neurovascular bundles were ligated prior to transection using 4-0 or 5-0 polyglactin 910 suture material. An 8 mm osteotome and 100 g mallet were then used to sever the mandibular symphyseal fibrocartilage and separate both mandibles. A periosteal elevator was used to elevate all remaining soft tissue and muscle attachments from the mandibular body. Care was taken to avoid laceration of the facial artery and vein crossing ventral and rostral to the angular process of the mandible. Gentle lateral traction of the affected mandible was applied to allow visualization of the mandibular foramen; the inferior alveolar nerve and artery were identified and ligated prior to transection. Ligation was performed using a pre-loaded single use hemoclip applicator. Based on the size of the inferior alveolar neurovascular bundle, the appropriate size of hemoclip (small, medium, or large) was selected. The jaws of the instrument were carefully positioned over the bundle and the hemoclip squeezed tightly into position. Two to three hemoclips were secured and carefully checked prior to incising the neurovascular bundle between 2 clips.

**Figure 2:**
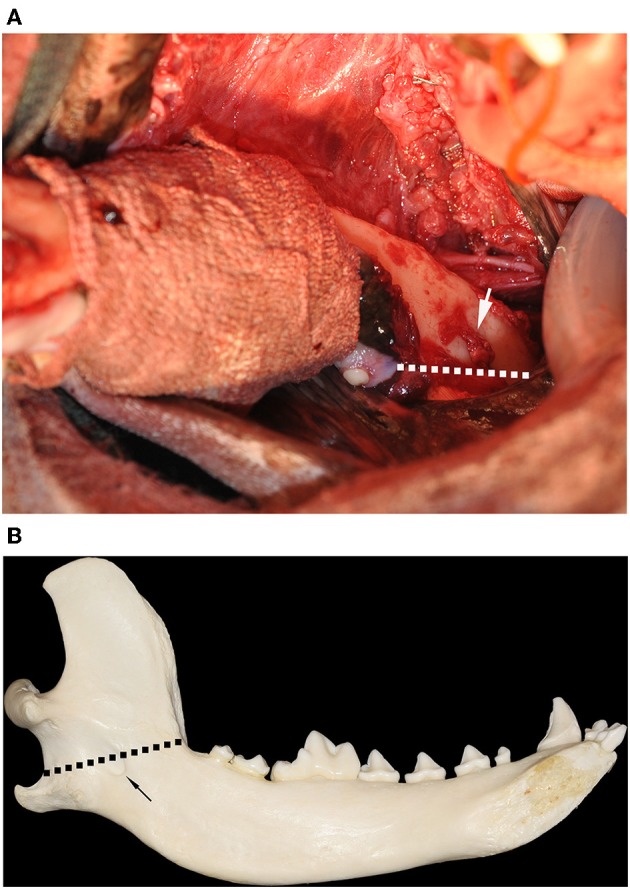
**(A)** Intraoperative clinical image of a left mandibular extended subtotal mandibulectomy with the patient in dorsal recumbency. The soft tissue dissection has been performed and the mandibular symphysis has been separated, allowing for lateralization of the jaw. The region of the gross tumor has been wrapped in sterile bandage material to prevent tumor cell dissemination during surgical manipulation. The inferior alveolar neurovascular bundle has been ligated and resected at the level of the mandibular foramen (white arrow). The planned osteotomy (broken white line) commenced distal to the left mandibular 3rd molar tooth and extended to the angle of the mandible, ensuring that the mandibular foramen is included in the excision. **(B)** Medial view of a dog's mandible showing the location of the osteotomy (broken black line) relative the mandibular foramen (black arrow).

**Figure 3:**
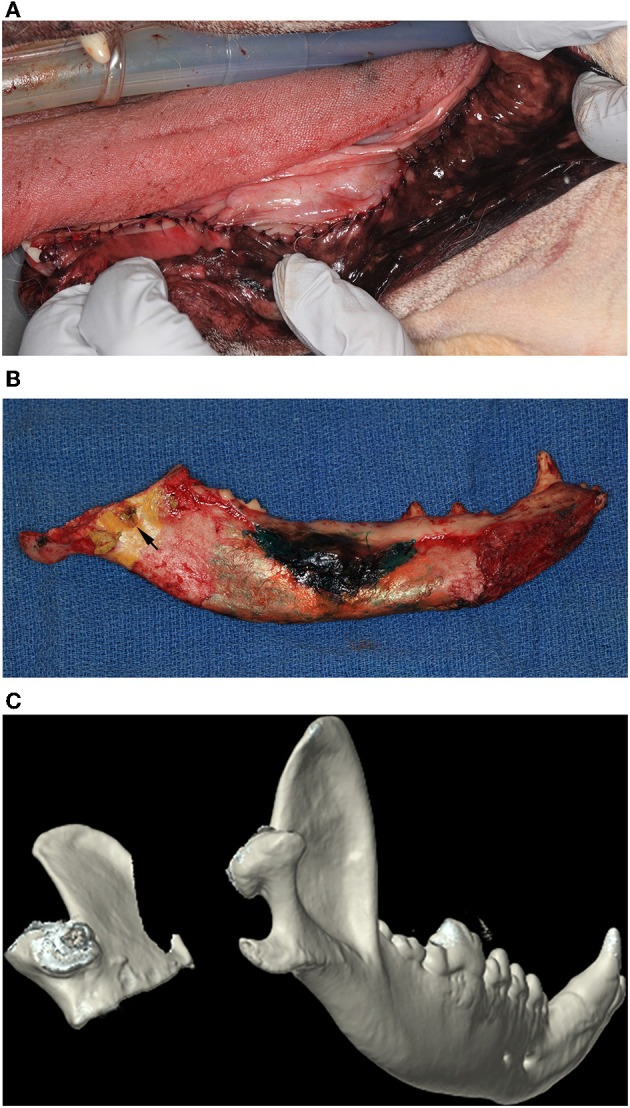
**(A)** Intraoral postoperative clinical image of the soft tissue repair following an extended subtotal mandibulectomy. **(B)** Postoperative clinical image of the medial aspect of a left mandible that has undergone an extended subtotal mandibulectomy. Note that the osteotomy included the mandibular foramen (black arrow). **(C)** Three-dimensional rendering of a postoperative CT of a dog that has undergone a left sided extended subtotal mandibulectomy. The left coronoid and condylar processes are conserved.

Once the soft tissue dissection was completed and the inferior alveolar neurovascular bundle was ligated, lateral traction on the mandible offered good visualization of the medial aspect of the jaw. Osteotomy was performed using a straight bone-cutting tip on a piezoelectric surgical handpiece (10), starting distal to the mandibular third molar tooth and extending caudally and dorsoventrally, ensuring that the mandibular foramen and the angular process remained entirely within the excised portion of the mandible (Figure 2). In one case, the patient was large, making access to the most caudal aspect of the mandible difficult to reach with the bone cutting instrument. In that case, an osteotome and mallet were carefully used to complete the osteotomy. The surgical sites were thoroughly lavaged with sterile 0.9% saline solution prior to routine apposition of the muscle and mucosal layers in a simple interrupted pattern using 5-0 poliglecaprone 25 in small breed dogs or 4-0 poliglecaprone 25 in medium to large breed dogs (Figure 3).

## Case Reports

### Case 1

A 13-year-old male castrated Belgian Tervuren dog was presented to the Dentistry and Oral Surgery Service at the Cornell University Hospital for Animals (CUHA) for treatment of a right mandibular osteosarcoma. On oral examination the mass was noted to extend from the level of the right mandibular third premolar tooth to the first molar tooth. Tumor staging was performed via mandibular lymph node fine needle aspirates and thoracic and abdominal computed tomographic (CT) images. No evidence of regional or distant metastasis was noted. A CT scan of the head was performed at the same time and this found a mass measuring 16 × 40 × 26 mm and extending form the distal aspect of the right mandibular third premolar tooth to the mesial root of the first molar tooth. The right mandibular fourth premolar tooth was missing. The mass was largely on the dorsal and buccal aspect of the mandible and resulted in lysis of the underlying alveolar bone. The lytic process extended into the mandibular canal.

Based on the clinical findings and the confirmed histological diagnosis of osteosarcoma, a right-sided extended subtotal mandibulectomy was performed. A surgical soft tissue margin of 15–30 mm was obtained around the gross tumor, including a skin wedge on the buccal aspect of the neoplasm. No surgical complications were encountered. Histological evaluation of the excised tissues later confirmed an osteosarcoma with tumor-free margins.

The patient recovered uneventfully from general anesthesia and was monitored overnight for evidence of hemorrhage and assessed for signs of pain. A constant rate infusion (CRI) of fentanyl (3 μg/kg/h); as well as meloxicam (0.1 mg/kg, IV, q24 h), and ampicillin/sulbactam (22 mg/kg, IV, q8 h) were also administered. The patient ate well overnight and appeared comfortable. The patient was discharged to the care of its owners the following day with a fentanyl patch (50 μg/h), meloxicam (0.1 mg/kg, PO, q 24 h), gabapentin (13 mg/kg, PO, q8–12 h), and amoxicillin/clavulanic acid (16 mg/kg, PO, q12 h). The owners were instructed to feed the patient a soft food diet and prevent any access to chews or toys for 3 weeks.

Due to geographic distance, the patient was examined by the referring veterinarian 2 weeks postoperatively. According to medical records available, the surgical site had healed and the patient was reported to be considerably more comfortable, playful, and had regained a historical voracious appetite. A 1-month postoperative examination with the referring veterinarian revealed that the patient was comfortable with no gross evidence of reoccurrence of the oral tumor. Based on a follow-up phone call with the owner, the patient continued to be in good health with no evidence of recurrence of the tumor for another 19 months before being euthanized due to unrelated disease.

### Case 2

A 9-year-old male castrated Labrador retriever dog was presented to CUHA's Dentistry and Oral Surgery Service for treatment of a left mandibular osteosarcoma located in the premolar and molar region. Tumor staging was performed via left and right mandibular lymph node fine needle aspirates and a thoracic and abdominal CT scan. No evidence of regional or distant metastasis was noted. A head CT scan performed at the same time found a mass measuring 23 × 55 × 10 mm and extended from the level of the left mandibular third premolar tooth to the mesial aspect of the second molar tooth. The left mandibular fourth premolar and first molar teeth were missing. Bone lysis was evident along the dorsal surface of the mandible and invasion of the mandibular canal was noted at the level of the tumor.

Given the location and extent of the tumor, a left-sided extended subtotal mandibulectomy was performed. A surgical soft tissue margin of 15–20 mm was obtained around the gross tumor. No surgical complications were encountered. Histopathological assessment of the surgical specimen showed complete excision of the osteosarcoma.

The patient recovered uneventfully from general anesthesia and was monitored overnight for evidence of hemorrhage, was assessed for signs of pain, and was maintained on intravenous fluids and administered a CRI of fentanyl (3 μg/kg/h) and dexmedetomidine (0.5 μg/kg/h), as well as ampicillin/sulbactam (22 mg/kg, IV, q8 h). The patient ate well overnight and appeared comfortable. The patient was discharged to the care of the owners with gabapentin (5 mg/kg q8–12 h), carprofen (2 mg/kg q12 h), and amoxicillin/clavulanic acid (20 mg/kg, PO, q12 h). The wonders were instructed to feed the patient a soft food diet and prevent any access to chews or toys for 3 weeks.

The patient was assessed by the primary care veterinarian 2 weeks postoperatively and then again at our facility ~1 month postoperatively. The surgical site had healed well, and the patient was active and eating without difficulty. Right-sided mandibular drift and occlusal trauma caused by the right mandibular canine tooth to the palate was noted. A crown-height reduction and endodontic therapy of the canine tooth were offered. However, the owner declined due to financial constraints. Follow-up with an oncologist was recommended for ongoing monitoring. The owner was contacted again 8 months following the procedure and reported that the patient continued to do well with no evidence of tumor recurrence.

### Case 3

A 9-year-old male castrated Pug dog was referred to CUHA's Oncology Service for evaluation of an incompletely excised oral melanoma at the level of the right mandibular third premolar tooth. On oral examination, no gross disease was evident. However, a scar and some suture material from the biopsy site were noted at the gingival margin at the level of the right mandibular second and third premolar teeth. The patient underwent tumor staging including an abdominal ultrasound, thoracic radiographs, and fine needle aspirates of the mandibular lymph nodes. No evidence of metastasis was found. A CT of the head was obtained and showed alveolar bone lysis and invasion of the tumor into the mandibular canal between the right mandibular first and third premolar teeth. The left and right mandibular lymph nodes were subjectively enlarged. Surgical excision and immunotherapy were elected as the treatment of choice and the patient was referred to the Dentistry and Oral Surgery Service.

A right-sided extended subtotal mandibulectomy was performed. A surgical soft tissue margin of 10–20 mm was obtained around the visible scar. The left and right mandibular lymph nodes were also surgically excised and submitted for histopathological analysis. Histopathological assessment of the submitted mandibular tissues showed a narrow (2 mm) margin at the ventral aspect of the soft tissue margin rostrally. All other aspects of the resected mandible were tumor-free. Both the left and right mandibular lymph nodes were found to be reactive and contain draining melanocytes and melanophages. However, the neoplastic nature of the melanocytes was not clear to the pathologists.

The patient recovered uneventfully from general anesthesia and was monitored overnight for evidence of hemorrhage and assessed for signs of pain. Intravenous fluids and a CRI of fentanyl (3 μg/kg/h) and dexmedetomadine (0.5 μg/kg/h) were administered, as well as meloxicam (0.1 mg/kgm, IV, q24 h), and ampicillin/sulbactam (22 mg/kg, IV, q8 h). The patient was comfortable overnight and ate well when hand-fed. The patient was discharged to the care of the owners the following day with meloxicam (0.1 mg/kg, PO, q24 h), pregabalin (2 mg/kg, PO, q12 h), and amoxicillin/clavulanic acid (15 mg/kg, PO, q12 h). The owners were instructed to feed the patient a soft food diet and prevent any access to chews or toys for 3 weeks.

The patient was examined 2-weeks postoperatively and was found to have healed well. A mild left-sided mandibular drift and tongue protrusion were noted. However, due to the patient's brachycephalic confirmation, there was no occlusal trauma. The Oncology Service commenced immunotherapy and monitoring. Eight-months postoperatively, recurrence of a pigmented mass in the rostral oral soft tissues, at the level of the previously reported narrow margin, was noted. The patient underwent a fine needle aspirate of the mass which was found to be a recurrence of the oral melanoma. Thoracic radiographs were obtained and no evidence of metastases noted. A full-thickness soft tissue excision with a 10 mm gross margin around the visible tumor was performed. The retropharyngeal lymph nodes were removed and submitted for histopathological analysis. Histopathological evaluation found tumor-free margins around the excised oral soft tissues. However, metastasis to the retropharyngeal lymph nodes was confirmed. The patient was examined 2-weeks postoperatively. The oral cavity had healed and the patient was reported to be comfortable and eating well. The patient returned to the Oncology Service for further monitoring and immunotherapy.

## Discussion

In this report we describe the technical aspects of an extended subtotal mandibulectomy that can be applied for the treatment of invasive mandibular body tumors that have extended into the mandibular canal in dogs, and illustrate its application using three clinical cases.

Excision of the mandibular body, from the level of the mental foramen to the mandibular foramen, is the accepted treatment modality for oral squamous cell carcinoma (OSCC) that has invaded the mandibular canal in human patients (11–13). The anterior and posterior perineural spread of OSCC in humans has been shown histologically to extend beyond the margins of the visible tumor but not beyond the bony confines of the nerve (11, 12). Based on this, it is common for veterinary oral surgeons to recommend a total mandibulectomy in dogs with oral tumors that have invaded the mandibular canal (6, 9). However, to the authors' knowledge, no studies looking at the rostral or caudal extension of invasive oral tumors that have extended into the mandibular canal in the dog, have been performed. Although it is reasonable to assume that a tumor would extend more readily along the path of least resistance, further investigation into mandibular canal extension of the various invasive canine oral neoplasms is warranted. Until that time, the current accepted standard is complete excision of the canal by means of a total mandibulectomy.

The surgical approach used to perform a total mandibulectomy in dogs is invasive, technically demanding and time consuming. It consists of the removal of the mandibular body and the ramus, and requires disarticulation of the temporomandibular joint. Additionally, extensive dissection of the insertions of the muscles of mastication (i.e., masseter, temporal, medial and lateral pterygoid, and digastricus) is required. With appropriate case selection, the described extended subtotal mandibulectomy technique achieves the same goal of complete mandibular canal resection. In addition, the procedure is considerably less invasive and time consuming, potentially reducing patient morbidity. Exact surgical time to complete this procedure was not included in this report due to a number of variables that were not controlled including; tumor size, complexity of the dissection, experience of the surgeon, small sample group, and the lack of a control group for comparison. Finally, the risk of hemorrhage from inadvertent trauma to the maxillary artery during temporomandibular joint disarticulation is eliminated (9). Based on the cases reported here, the only postoperative complications encountered were mandibular drift and tongue protrusion (14–16). These are also common in cases that have undergone total mandibulectomy (6, 9, 14).

Appropriate patient selection is critical when considering an extended subtotal mandibulectomy. Diagnostic imaging to assess the size, location, and extent of a tumor, in particular with reference to the mandibular canal and the ramus, is an essential step when determining the most appropriate surgical procedure. Computed tomography is considered the gold standard of diagnostic imaging of neoplasms affecting the maxillofacial region in cats and dogs (17). The extended subtotal mandibulectomy is intended for cases where invasive tumors are growing in the mandibular body and invading the mandibular canal. For mandibular body tumors that are not invading the mandibular canal, a subtotal, segmental, or marginal excision may be more appropriate (2, 6, 9). On the other hand, a total mandibulectomy is the treatment of choice for tumors that involve both the mandibular body and the ramus.

The three cases described here had tumors of the mandibular body with clear invasion of the mandibular canal. Two of the cases were presenting with osteosarcomas and one case with oral melanoma. Both malignancies are locally invasive and destructive. The extended subtotal mandibulectomy was found to achieve the goal of excising *en bloc* the entire mandibular canal while reducing surgical trauma and operating time. Although case number 3 had local reoccurrence of the oral melanoma, likely due to a narrow soft tissue margin, none of the cases had evidence of recurrence of the tumor more caudally, at the level of the mandibular foramen. Postoperative histological assessment of the excised tissues was only performed at the surgical margins. Future studies assessing the extent of various tumors along the mandibular canal, including perivascular and perineural involvement, would be of clinical importance in the future.

We propose that the extended subtotal mandibulectomy is a viable surgical technique that allows for adequate *en bloc* excision of mandibular body tumors invading the mandibular canal. The technique, when applied to the appropriate case, provides the surgeon with the ability to adhere to sound oncological principles while reducing the necessity of the more invasive total mandibulectomy technique.

## Data Availability Statement

All datasets generated for this study are included in the manuscript/supplementary files.

## Ethics Statement

The use of archived diagnostic material or review and data collection from medical records of client-owned animals for the purposes of this study was approved by Cornell University's Veterinary Clinical Studies Committee and was considered exempt from review by Cornell University's Institutional Animal Care and Use Committee.

## Author Contributions

NF and SP contributed equally to the concept of the manuscript. The writing was primarily by NF.

### Conflict of Interest

The authors declare that the research was conducted in the absence of any commercial or financial relationships that could be construed as a potential conflict of interest.
